# Changes in glycosylated haemoglobin and treatment outcomes in patients with tuberculosis in Iran: a cohort study

**DOI:** 10.1186/s40200-014-0123-0

**Published:** 2014-12-16

**Authors:** Payam Tabarsi, Parvaneh Baghaei, Majid Marjani, William M Vollmer, Mohammad- Reza Masjedi, Anthony D Harries

**Affiliations:** Clinical Tuberculosis and Epidemiology Research Center, National Research Institute of Tuberculosis and Lung Diseases (NRITLD), Masih Daneshvari Hospital, Shahid Beheshti University of Medical Sciences, Tehran, Iran; Kaiser Permanente Center for Health Research, Portland, OR USA; Chronic Respiratory Disease Research Center, National Research Institute of Tuberculosis and Lung Diseases (NRITLD), Shahid Beheshti University of Medical Sciences, Tehran, Iran; International Union Against Tuberculosis and Lung Disease, Paris, France; London School of Hygiene & Tropical Medicine, London, UK

**Keywords:** Glycosylated haemoglobin (HbA1c), Tuberculosis, Outcome, Temporary hyperglycemia

## Abstract

**Background:**

Diabetes mellitus (DM) affects tuberculosis (TB) treatment outcomes, mostly by increasing recurrence, mortality and treatment failure. The objectives were to determine the pattern of change in glycosylated haemoglobin (HbA1c) level in new TB patients admitted to hospital at the start and 3-months after TB treatment, and to relate the measurements at these two time intervals to whether patients successfully completed treatment.

**Methods:**

A prospective cohort study was conducted on hospitalized new TB patients at Masih Daneshvari Hospital from 2012 to 2013. All patients were tested for HbA1c at the beginning and 3 months after initiation of TB treatment. Changes in HbA1c were compared to TB treatment outcome.

**Results:**

There were 317 new TB cases admitted to hospital of which 158 had HbA1c at baseline and 3-months. Of these, 67 (42%) had normal values, 54 had an elevated HbA1c at either base-line or 3-months (uncertain diabetes status) and 37 (24%) had elevated HbA1c (≥6.5%) at both time points (DM). There were differences between the groups: those with DM were older, had a known history of DM and a higher prevalence of cavities on chest x-ray. There were 150 (95%) patients who successfully completed treatment with no significant differences between the groups.

**Conclusion:**

There were changes in HbA1c during the first three-months of anti-TB treatment, but these were not associated with differences in TB treatment outcomes. Transient hyperglycemia should be considered in TB patients and needs to be taken into account in planning care and management.

## Background

The risk of tuberculosis (TB) is increased in patients with diabetes mellitus (DM) [1,2]. The evidence for this association between DM and TB mainly comes from industrialized countries, although increasingly the association is being shown in other countries of the world [3]. The association between the two diseases is important as DM affects TB treatment outcomes, mainly by delaying sputum culture conversion, increasing case fatality and treatment failure, and increasing the risk of recurrent TB after completion of anti-TB treatment [4].

The global consequences of the interaction between DM and TB are compounded by the fact that there is a global epidemic of DM, with prevalent numbers expected to rise from 370 million in 2012 to over 550 million by 2030 [3]. Iran is similarly affected by a DM epidemic. The prevalence of DM in the general population aged above 40 years is estimated at 24%, and 10 million DM patients are estimated to be living now in Iran [5].

It is recognized that blood glucose levels can temporarily increase as a result of infection with TB, which is a chronic infectious disease [6]. This interaction, and the rise in blood glucose levels, can result in false positive diagnoses of DM if blood glucose levels are measured at the time of registration of TB patients [7,8]. There are limited data to show whether blood glucose levels stay the same, decrease or even increase with TB treatment and whether there are any associations between these changes in blood glucose levels and final TB treatment outcomes.

In Iran, we have been assessing blood glucose status by measurement of glycosylated haemoglobin (HbA1c). Although there are some challenges with measuring HbA1c, this is a better marker of blood glucose control as it provides a measure of blood glucose over a period of 2–3 months and is not subject to the rapid swings that can occur with random and fasting blood glucose measurements [9]. We therefore decided to examine HbA1c measurements in new TB patients admitted and treated at the National Research Institution of Tuberculosis and Lung Diseases (NRITLD), Masih Daneshvari Hospital, in Tehran, Iran, at the start and 3-months after TB treatment, and to relate the measurements at these two time intervals to whether the patients successfully completed treatment.

## Methods

### Design and setting

A prospective cohort study of newly TB diagnosed patients who were admitted at NRITLD was carried out from May 2012 to May 2013. This institute is one of the World Health Organization (WHO) collaborating centers for the Eastern Mediterranean Region (EMRO) and has been certified as the National Referral Laboratory.

### Management of patients

Patients with symptoms and signs suggestive of TB are referred to NRITLD where a diagnosis of TB is made on the basis of a positive smear for acid fast bacilli (AFB), a culture of *Mycobacterium tuberculosis*, a rapid nucleic acid amplification test (PCR) or histopathology showing characteristic pathological findings. Specimens with positive cultures are also assessed by drug sensitivity testing. Patients with a diagnosis of TB are treated based on national and international guidelines for 6 months with standard drugs (isoniazid, rifampicin, pyrazinamide and ethambutol for 2 months followed by isoniazid and rifampicin for 4 months) or for longer if it is necessary [10]. If drug-resistance was identified and especially resistance to isoniazid and rifampicin, the treatment regimen was changed accordingly and in line with national guidelines. Baseline laboratory evaluation includes measurement of random and fasting blood glucose (RBG and FBG), HbA1c, and other laboratory tests as necessary. HbA1c was measured using a Nycocard® HbA1c reader on whole blood with a printed range of values from 4%–15% (±0.1). All patients were also tested for HIV using rapid diagnostic tests. TB patients with HbA1c ≥6.5% are managed in consultation with a nutritionist and referred to Diabetes care for control of their diabetes.

After discharge from the hospital, treatment is continued at peripheral health centers and it is also recommended that patients return to hospital after 3 months for a second measurement of HbA1c.

For all patients, sputum smear and culture are repeated at month 5 and at the end of treatment to evaluate whether there is treatment failure. The outcome of TB treatment is defined according to WHO guidelines [10]. We considered cured and completed treatment as TB treatment success and all other outcomes such as failure, default, transferred out and all-cause mortality as adverse outcomes.

### Patient sample

All adult patients aged above 15 years who were hospitalized with newly diagnosed TB were enrolled in the study. This included all pulmonary TB patients with a positive sputum smear for acid-fast bacilli (AFB) or a positive culture of *Mycobacterium tuberculosis.* It also included all extra-pulmonary TB patients with positive histopathology, PCR, AFB smear or culture of a sample obtained from the site of disease. HbA1c was assessed at the time of registration and 3-months later, and only patients who had both these measurements were included in the study assessment.

### Data variables, data sources and data collection

Patient data were obtained from a patient TB register into a paper-based proforma. Data variables included age, sex, a new or known diagnosis of DM, smoking status, drug abuse history, duration of symptoms, type of TB, cavitary lesion in chest x-ray, grade of sputum smear at base line, FBG level, HbA1c level, and final TB treatment outcomes. The grade of sputum smear at base line was defined as: 10–99 bacilli in 100 high power field = 1^+^; 1–10 bacilli in one high power field = 2^+^; more than 10 bacilli in one high power field = 3^+^; scanty as less than 10 bacilli in 100 high power fields. These data were single entered into SPSS version 11.5 [SPSS Inc, Chicago, IL, USA]. All records were checked for completeness, reliability and precision.

### Data analysis and statistics

All demographic and clinical information of TB patients were transported from SPSS to STATA and analyzed using Stata, version 12.1 (Statacorp. College Station, Texas, USA). Patients with TB were divided into four groups based on the measurements of HbA1c at the two different time points:

1. Normal- Normal (NN): The patients who had HbA1c less than 6.5% at two time points. They were defined as normal
2. Elevated- Normal (EN): The patients who had HbA1c ≥6.5% at the baseline and at the third month had <6.5%. They were defined as uncertain DM status.
3. Normal- Elevated (NE): The patients who had HbA1c <6.5% at baseline and after 3 months the level was more than 6.5%. They were defined as uncertain DM status.
4. Elevated- Elevated (EE): The patients who had HbA1c ≥ 6.5% at the beginning and after 3 months of treatment. They were defined as abnormal and having DM.

The characteristics and treatment outcome of these four groups were compared. We eliminated the normal-elevated group from formal significance testing due to the limited number of subjects (n = 5) who fell into this group. Categorical variables were compared by using Chi-square or Fisher’s exact test and continuous variables using ANOVA. All reported p values are two-sided.

### Ethics

The study protocol was reviewed and approved by the NRITLD Scientific and Ethics Committee, Teheran, Iran.

## Results

There were 525 new TB patients aged above 15 years who presented to NRITLD between May 2012 and May 2013. Of these, 208 patients refused to be hospitalized and were referred to peripheral health centers to receive treatment, leaving 317 confirmed TB cases with median age 58 years (inter-quartile range [IQR] 35.5–73.5) that were admitted to hospital. There were 158 patients who had HbA1c at baseline and 3 months and 159 patients who only had HbA1c performed as baseline. The characteristics of these two groups of patients are shown in Table 1. The two groups were similar except that those who had both baseline and 3-month HbA1c were younger than those who had only HbA1c at baseline.

**Table 1:** **Characteristics of TB patients in relation to HbA1c at baseline and at baseline- 3 months**

**Characteristics**	**All hospitalized patients**	**Patients with HbA1c level at baseline and at 3-months**	**Patients with only baseline HbA1c level**	**p-value** ^**†**^
Number of patients	317	158	159	
Age (Mean ± SD) yrs	55.33 ± 21.04	52.83 ± 21.24	57.82 ± 20.60	0.03
Male gender	163 (51.4%)	77 (48.73%)	86 (54.10%)	0.37
History of smoking	96 (30.3%)	44 (27.85%)	52 (32.70%)	0.40
History of opium use	74 (23.3%)	31 (19.62%)	43 (27%)	0.14
Positive HIV test	17 (5.4%)	5 (3.20%)	12 (7.50%)	0.13
Diabetes mellitus (known)	46 (14.5%)	22 (13.92%)	24 (15.10%)	0.87
Pulmonary tuberculosis	296 (93.4%)	151 (95.57%)	145 (91.20%)	0.20
Symptom duration (months)	3.89 ± 3.18	3.90 ± 3.37	3.88 ± 2.99	0.94
Smear grade of sputum				
3+	114 (36%)	59 (37.34%)	55 (34.6%)	0.79
2+	32 (10.1%)	13 (8.23%)	19 (11.9%)	
1+	77 (24.3%)	38 (24.05%)	39 (24.5%)	
Scanty	44 (13.9%)	24 (15.20%)	20 (12.6%)	
Negative	50 (15.8%)	24 (15.20%)	26 (16.4%)	
HbA1c (baseline)	7.2 ± 2	7.2 ± 2	7.1 ± 2	0.37
FBG (baseline)	119.1 ± 51.1	119 ± 53	119.3 ± 49.3	0.94
Cavitary lesion	155 (48.9%)	75 (47.50%)	80 (50.3%)	0.65

^**†**^Student’s t-test and x^2^ test or Fisher exact is used when appropriate.

*Abbreviations:* *TB* tuberculosis, *HbA1c* Glycosylated haemoglobin, *SD* Standard deviation, *HIV* human immunodeficiency virus, *FBG* Fasting blood glucose.

Of 158 patients with HbA1c at baseline and at 3-months, there were 67 (42%) with normal values at both time points, 5 (3%) with a normal baseline and elevated 3-month value, 49 (31%) with an elevated HbA1c at baseline and normal value at 3-months and 37 (24%) with elevated HbA1c at both time points. Thus, 54 (34%) patients had an uncertain DM status and 24% had DM. Baseline characteristics of patients with HbA1c done at baseline and at 3-months are shown in Table 2. There were some differences between the groups with age, a known history of DM and prevalence of cavitatory lesions being highest in patients with DM, and a history of smoking being highest in those with normal glucose status. Baseline HbA1c and FBG were highest in those with confirmed DM.

**Table 2:** **Characteristics of TB patients who had both HbA1c done at baseline and 3-months**

**Characteristics**	Patients with HbA1c level at baseline and 3-months* N = 158				**p- value** ^**†**^
	**Normal-Normal**	**Normal–Elevated**	**Elevated-Normal**	**Elevated-Elevated**	
Number of patients	67	5	49	37	
Age (Mean ± SD) yrs	45.3 ± 22.3	58.2 ± 19.8	54.9 ± 21.2	62.9 ± 13.6	0.001
Male gender	34 (50.7%)	2 (40%)	22 (44.9%)	19 (51.4%)	0.78
History of smoking	25 (37.3%)	0	8 (16.3%)	11 (29.7%)	0.05
History of opium use	15 (22.4%)	1 (20%)	8 (16.3%)	7 (18.9%)	0.71
Positive HIV test	3 (4.5%)	0	2 (4.1%)	0	0.43
Diabetes mellitus(known)	1 (1.5%)	0	1 (2%)	20 (54.1%)	0.001
Pulmonary tuberculosis	61 (91%)	5 (100%)	49 (100%)	36 (97.3%)	0.06
Symptom duration (months)	3.64 ± 3.38	5 ± 2.35	4.13 ± 3.72	3.92 ± 3.02	0.75
Smear grade					0.42
3+	22 (32.8%)	4 (80%)	17 (34.7%)	16 (43.2%)	
2+	7 (10.4%)	0	3 (6.1%)	3 (8.1%)	
1+	13 (19.4%)	0	16 (32.7%)	9 (24.3%)	
Scanty	10 (14.9%)	0	9 (18.4%)	5 (13.5%)	
Negative	15 (22.4%)	1 (20%)	4 (8.2%)	4 (10.8%)	
Cavitary lesion	29 (43.3%)	3 (60%)	19 (38.8%)	24 (64.9%)	0.04
HbA1c (baseline) (%)	5.9 ± 0.4	6.1 ± 0.3	7.1 ± 1	10 ± 2	0.01
FBG (baseline) (mg/dl)	96.1 ± 14.4	118.8 ± 34	104.6 ± 32.5	179.1 ± 73.1	0.001

*Normal level of HbA1c is considered less than 6.5% and elevated level is considered more than 6.5%.

**†** Student’s t-test and x^2^ test or Fisher exact is used when appropriate.

*Abbreviations:* *TB* tuberculosis, *HbA1c* Glycosylated haemoglobin, *SD* Standard deviation, *HIV* human immunodeficiency virus, *FBG* Fasting blood glucose.

There were 150 (95%) patients who successfully completed treatment, with four patients failing treatment, one lost to follow-up (defaulted) and three having died. Treatment outcomes in relation to HbA1c levels at baseline and at 3-months are shown in Table 3. There were no significant differences between the different groups.

**Table 3:** **Relationship of HbA1c levels at baseline and 3-months with final TB treatment outcomes**

**Outcome of TB treatment**	Patients with HbA1c level at baseline and 3-months*				**p-value**
	**Normal-Normal**	**Normal–Elevated**	**Elevated-Normal**	**Elevated-Elevated**	
Number of patients	67	5	49	37	
Number (%) with treatment success	64 (95.5%)	5 (100%)	46 (93.9%)	35 (94.6%)	0.97
Number (%) failing treatment	2 (3%)	0	1 (2%)	1 (2.7%)	
Number (%) lost to follow-up (defaulted)	0	0	1 (2%)	0	
Number (%) who died	1 (1.5%)	0	1 (2%)	1 (2.7%)	

*Normal level of HbA1c is considered less than 6.5% and elevated level is considered more than 6.5%.

*Abbreviations:* *TB* tuberculosis, *HbA1c* Glycosylated haemoglobin, *SD* Standard deviation, *HIV* human immunodeficiency virus, *FBG* Fasting blood glucose.

## Discussion

This study in Iran shows that of patients admitted to hospital with pulmonary or extra-pulmonary TB, just over 40% had normal HbA1c at baseline and three months, 24% had DM and the remainder had an uncertain DM status based on one elevated HbA1c either at baseline or 3-months into treatment. There were some differences in baseline characteristics between the four groups, with patients who had definite DM being older and having a higher prevalence of pulmonary cavitary lesions on chest x-ray and also having the highest levels of fasting blood glucose and HbA1c. Treatment outcomes for the whole group were good with nearly 95% achieving treatment success, and there were no differences between any of the groups.

The strengths of the study were that unselected consecutive patients were enrolled in the study, all patients had confirmed TB and were treated and monitored in the same way, and the baseline characteristics, apart from a slight difference in age, of those with two HbA1c measurements were similar to those who only had one baseline measurement. Thus, the findings are probably representative of all patients admitted to the hospital. Limitations relate to the fact that the study was carried out in a referral center, so we cannot claim that our findings are representative of all TB patients with or without DM in Iran.

In nearly one third of patients, the high HbA1c level at baseline was recorded as normal at 3-months. In studies using fasting blood glucose measurements, the so called “temporary hyperglycemia” (TH) is well recognized [11] and several studies have reported glucose intolerance at the initiation of TB treatment followed by normalization during treatment [7,12,13]. This may be due to the general inflammatory status associated with active TB, but if blood measurements are carried out shortly after registration and initiation of anti-TB treatment, then this may also be due to the hyperglycemic effects of rifampicin and isoniazid [14]. Whatever the precise reason, this is an important finding and suggests that a firm diagnosis of DM should not be made on just one baseline result, whether a fasting blood glucose or HbA1c is used.

A recent systematic review documented that mortality and failure rates are increased in patients with DM [4]. Other studies have confirmed these findings of higher failure rates [15] and higher rates of all-cause mortality [16,17]. Alisjahbana et al., showed that treatment failure among DM tuberculosis patients was higher than among non-DM tuberculosis patients after adjusting for non-compliance and drug resistance (odds ratio 7.65) [18]. However, this is not always the case. Some studies have shown no relationship between DM or poor glucose control and the outcome of TB treatment [19-23]. In a recent study in China, fasting blood glucose (FBG) was used to document DM status, and no association was found between TB treatment outcome and FBG [14]. A more recent study in a referral centre in India found no effect of DM on TB treatment outcomes; although there was a trend towards smear non-conversion at 2-months [24]. These are similar to the findings of our current study where we have found no association between glucose status and treatment outcomes. Differences in associations between DM and TB treatment outcomes may be due to a number of reasons. There is the power of the study, especially if the TB program has excellent treatment outcomes, which means there is a need for larger numbers of patients to ascertain cause and effect. Then there are issues such as confounders (for example, smoking and HIV status) and the levels of DM control at the start and during anti-TB treatment. All of these require further prospective study with large numbers of patients.

Although we raise questions about the timing of blood glucose measurement during TB treatment, we believe it is important to screen for DM near to the start of treatment if only to intervene and institute appropriate care to reduce hyperglycemia [25]. Even if this does not have an effect on TB treatment outcomes, and at present there are few published data on whether tight glucose control impacts on TB treatment outcomes, [6] it is good clinical practice.

## Conclusion

In summary, this study shows glycosylated haemoglobin levels can change during the first 3-months of anti-TB treatment, but whether glucose status was defined as normal, uncertain or abnormal this had no effect on TB treatment outcomes. Further studies are needed to determine the prevalence and importance of temporary hyperglycaemia (TH) in other communities.

## Abbreviations

TB: Tuberculosis
DM: Diabetes Mellitus
HbA1c: Glycosylated haemoglobin
NRITLD: National Research Institution of Tuberculosis and Lung Diseases
WHO: World Health Organization
EMRO: Eastern Mediterranean Region
AFB: Acid fast bacilli
FBG: Fasting blood glucose
RBG: Random blood glucose
PCR: Polymerase chain reaction
HIV: Human immunodeficiency virus
TH: Temporary hyperglycaemia
